# Routine use of intraoperative nerve monitoring is associated with a reduced risk of vocal cord dysfunction after thyroid cancer surgery

**DOI:** 10.1186/s12893-023-02122-3

**Published:** 2023-08-02

**Authors:** Alexander Wilhelm, Patricia C. Conroy, Lucia Calthorpe, Willow Frye, Julie Ann Sosa, Sanziana Roman

**Affiliations:** 1 Department of Surgery, University of California, San Francisco, San Francisco, CA USA; 2 Department of Surgery, Clarunis - St. Clara Hospital and University Hospital Basel, Basel, Switzerland

**Keywords:** CESQIP, Thyroid Cancer, Intraoperative nerve monitoring, Vocal cord dysfunction

## Abstract

**Background:**

The aim of this study was to investigate the associations between individual surgeon’s intraoperative nerve monitoring (IONM) practice and factors associated with vocal cord (VC) dysfunction in patients with thyroid cancer undergoing thyroidectomy.

**Methods:**

Using Collaborative Endocrine Surgery Quality Improvement Program (CESQIP) 2014-21 data, multivariable logistic regression analyses investigated variables associated with short- and long-term VC-dysfunction, associations of routine use of IONM with postoperative outcomes, and patient characteristics associated with IONM use.

**Results:**

Among 5,446 patients (76.7% female, mean age 49 years), 68.5% had surgery by surgeons using IONM in ≥ 90% of cases (63% of surgeons, n = 73). Post-operative VC-dysfunction was diagnosed by laryngoscopy in 3.0% of patients in the short-term and 2.7% in the long-term. When surgeons routinely used IONM, the incidence of VC-dysfunction was 2.4% in the short-term and 2.2% in the long-term, compared to 4.4% and 3.7%, respectively, when surgeons did not routinely use IONM (p < 0.01). After adjustment, routine use of IONM was independently associated with reduced risk of short- (OR 0.48, p < 0.01) and long-term (OR 0.52, p < 0.01) VC-dysfunction, a lower risk of postoperative hypoparathyroidism in the short- (OR 0.67, p < 0.01) and long-term (OR 0.54, p < 0.01), and higher likelihood of same-day discharge (OR 2.03, p < 0.01). Extrathyroidal tumor extension and N1-stage were factors associated with postoperative VC-dysfunction in the short- (OR 3.12, p < 0.01; OR 1.92, p = 0.01, respectively) and long-term (OR 3.11, p < 0.01; OR 2.32, p < 0.01, respectively).

**Conclusion:**

Routine use of IONM was independently associated with a lower risk of endocrine surgery-specific complications and greater likelihood of same-day discharge.

## Introduction

Recurrent laryngeal nerve (RLN) injury is a feared complication of thyroid and parathyroid surgery that has potentially devastating consequences. Transient or permanent RLN injury occurs in 1.1–11% of patients after thyroidectomy [1]. Unilateral vocal cord (VC) dysfunction after unilateral nerve injury can lead to hoarseness, while bilateral RLN injury can result in life-threatening aphonia possibly requiring intubation and tracheostomy [2]. Intraoperative nerve monitoring (IONM) has served as an adjunct to the gold standard of direct nerve visualization for prevention of RLN injury [3].

Although adoption of IONM has increased over time, several studies examining its use in thyroid surgery have failed to demonstrate a reduced risk of RLN injury with IONM [4, 5]. However, many of these studies were inadequately powered given the overall rarity of RLN injury [6–8]. According to the 2015 American Thyroid Association (ATA) guidelines, IONM “may be considered to facilitate nerve identification and confirm neural function” [9]. More recently, Kim et al. analyzed 17,160 patients in the American College of Surgeons National Surgical Quality Improvement Program (NSQIP) and found that IONM was associated with lower risk of RLN injury [1]. However, only a minority of patients in their cohort underwent thyroidectomy for thyroid cancer, no distinction was made between temporary and permanent VC dysfunction, and VC dysfunction was not confirmed on laryngoscopy [1, 10]. In addition, the benefit of IONM is unclear if surgeons are unfamiliar with the technique and use it only selectively. A previous study showed that the absence of standardization in the use of IONM represents the main cause of incorrect results, which may increase the risk of RLN injury due to misleading information [11].

In contrast to NSQIP, the Collaborative Endocrine Surgery Quality Improvement Program (CESQIP), which contains data from specialized thyroid surgeons and sites, also includes information on laryngoscopy-confirmed short- (≤ 30 days postoperatively) and long-term (31–180 days) VC dysfunction, and contains information on the individual surgeon’s IONM practice. This level of granularity is important to accurately understand the clinical impact of IONM and the surgeon’s IONM practice. The current study aimed to determine the potential association between IONM and post-operative VC dysfunction confirmed with laryngoscopy among patients with thyroid cancer, one of the groups at highest risk of RLN injury during thyroidectomy. Our hypothesis was that routine use of IONM would be associated with a lower risk of RLN injury among thyroid cancer patients.

## Methods

### Data source

CESQIP was founded in 2012 by endocrine surgery leaders from the American Association of Endocrine Surgeons (AAES) and began collecting data in 2014 [12]. The CESQIP Aggregate Data Program (CADP) is a de-identified database that contains data exclusively from specialized endocrine/thyroid surgeons and sites. The CESQIP database includes granular pathologic data, including thyroid cancer histology and AJCC/TNM pathologic staging, as well as endocrine surgery-specific outcome variables, including neck hematoma, vocal cord dysfunction, and postoperative hypoparathyroidism events.

### Study design

**Fig. 1:**
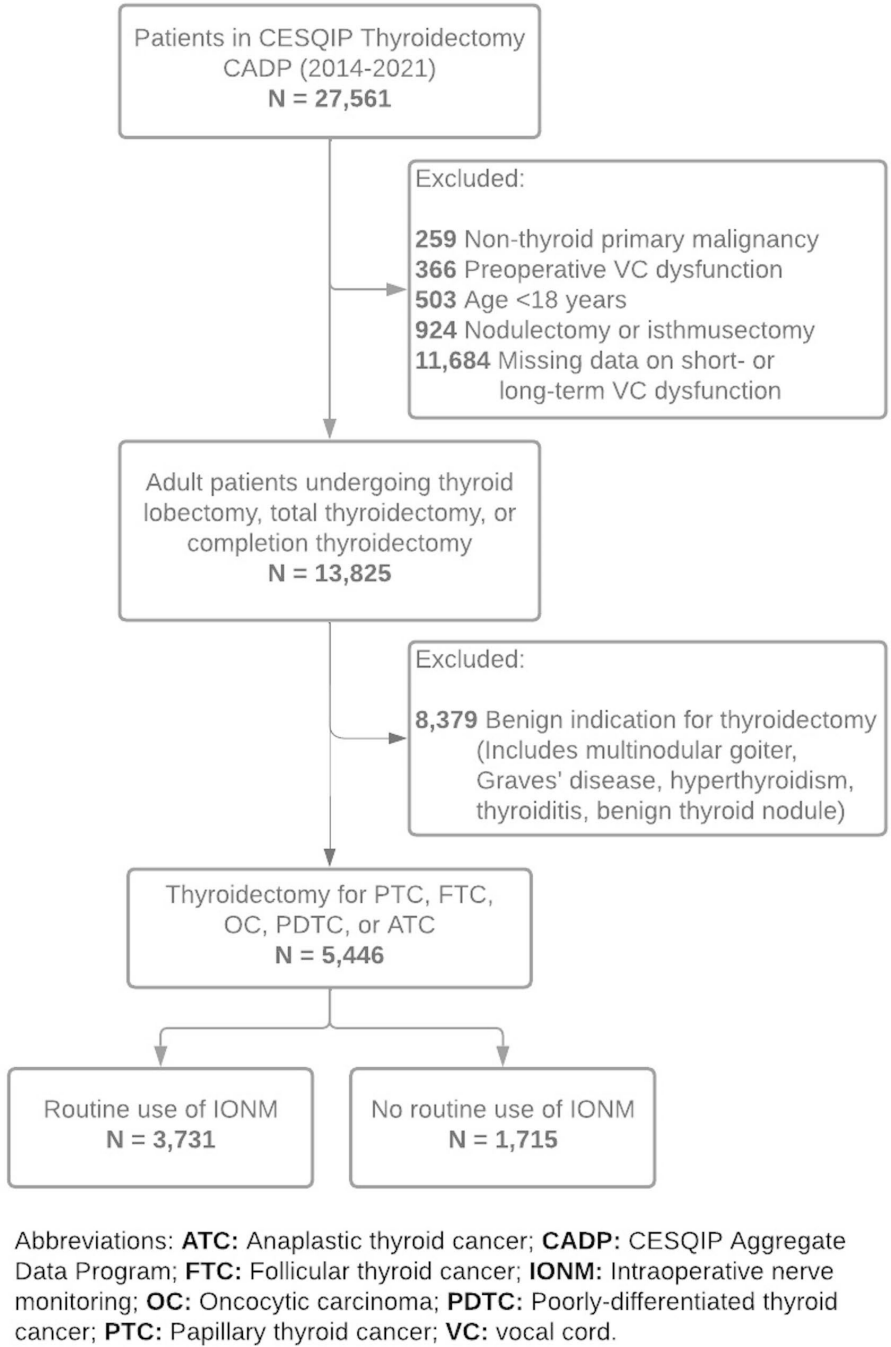
Participant flow diagram

Patients aged ≥ 18 years who underwent thyroidectomy for pathologically confirmed thyroid cancer between January 2014 and April 2021, captured in the CESQIP thyroidectomy CADP, were examined (Fig. 1). Papillary (PTC), follicular (FTC), oncocytic, medullary (MTC), poorly differentiated (PDTC), and anaplastic (ATC) thyroid cancer histologies were included. Patients with preoperative VC dysfunction and those with missing data on short- and/or long-term VC dysfunction were excluded. Routine use of IONM was defined as the use of nerve monitoring in ≥ 90% of each surgeon’s cases. The primary endpoints were postoperative VC dysfunction confirmed by laryngoscopy at ≤ 30 days (short-term) and 31–180 days (long-term) postoperatively. Secondary endpoints were postoperative hypoparathyroidism events (defined as therapeutic calcium and/or vitamin D supplementation or low calcium and/or parathyroid hormone (PTH) levels) in the short- (≤ 30 days postoperatively) and long-term (30–180 days postoperatively), length of hospital stay, and postoperative neck hematoma.

### Statistical analysis

Demographic, clinical, and pathological characteristics were compared between patients who underwent thyroidectomy by surgeons who used IONM routinely and those who did not by means of two-sided t-tests for continuous variables and χ^2^-tests for categorical variables. Multivariable logistic regression models were employed to examine associations between surgeon’s practice of IONM and postoperative outcomes. Covariables were selected based on previous studies and clinical judgement and included patient age, sex, race, body mass index (BMI), prior neck surgery, prior neck radiation, extent of surgery (thyroid lobectomy, total thyroidectomy, or completion thyroidectomy), concomitant central or lateral neck dissection, operative time, thyroid cancer histology, extrathyroidal tumor extension, pathologic N-stage, and postoperative neck hematoma. Sensitivity analyses included cut-off values of 80% and 75% defining routine IONM use. A two-sided alpha of 0.05 was considered statistically significant for all analyses. Statistical analyses were performed using Stata/BC version 16.1 (StataCorp LLC, College Station, TX). This study was granted an exemption by our Institutional Review Board due to use of de-identified data.

## Results

### Demographic, clinical, and pathological characteristics

Between January 2014 and April 2021, 5,446 patients in the CESQIP thyroidectomy CADP met inclusion criteria. Among these, most were female (76.7%), and the mean age was 49 years (SD = 15.3 years) (Table 1). PTC was the most common thyroid cancer histology (84.6%); 27.3% of patients had nodal metastases; and 3.9% had extrathyroidal tumor extension. Overall, IONM was used in 74.1% of cases. Among all surgeons in CESQIP, 63% (n = 73) used IONM in ≥ 90% of cases (“routine IONM use”); the remaining 37% (n = 44) of surgeons used IONM in less than 90% of their cases. The incidence of laryngoscopy-confirmed postoperative VC dysfunction was 3.0% for short-term and 2.7% for long-term. In patients operated on by surgeons who routinely use IONM, the incidence of VC dysfunction was 2.4% in the short-term and 2.2% in the long-term, compared to 4.4% and 3.7% respectively, for patients who underwent surgery by surgeons who do not routinely use IONM (p < 0.01). The highest proportions of postoperative VC dysfunction were observed in medullary thyroid cancer (MTC) (short-term VC dysfunction: 7.4%; long-term VC dysfunction: 5.2%) and poorly differentiated & anaplastic thyroid cancer (PDTC + ATC) (short-term VC dysfunction: 6.3%; long-term VC dysfunction: 6.3%) compared to PTC (short-term VC dysfunction: 2.9%; long-term VC dysfunction: 2.5%).

**Table 1:** Demographic and clinical characteristics of patients who underwent thyroidectomy for thyroid cancer by surgeons who use IONM in ≥ 90% of cases (routine IONM use) or less (no routine IONM use)

	No Routine IONM use (n = 1,715)	Routine IONM use (n = 3,731)	*P* value*
Patient Characteristics			
Female sex	1323 (77.1%)	2858 (76.6%)	0.66
Age, years, mean (SD)	48.8 (15.5)	49.6 (15.2)	0.12
Race			< 0.01
Black	52 (3.0%)	247 (6.6%)	
White	1037 (60.8%)	2509 (67.4%)	
Other	223 (13.1%)	399 (10.7%)	
Hispanic	330 (19.4%)	381 (10.2%)	
Asian	63 (3.7%)	186 (5.0%)	
BMI > 40	124 (7.3%)	336 (9.0%)	0.03
Pre-op compressive symptoms	242 (15.2%)	673 (19.9%)	< 0.01
Preoperative FNA Result			< 0.01
Bethesda 1	17 (1.2%)	43 (1.3%)	
Bethesda 2	103 (7.5%)	354 (11.0%)	
Bethesda 3	172 (12.5%)	676 (21.1%)	
Bethesda 4	233 (16.9%)	335 (10.5%)	
Bethesda 5	221 (16.1%)	478 (14.9%)	
Bethesda 6	629 (45.7%)	1318 (41.1%)	
Prior anterior neck surgery	114 (6.7%)	235 (6.3%)	0.60
Prior neck irradiation	33 (1.9%)	106 (2.8%)	0.05
Perioperative Characteristics			
Extent of Surgery			< 0.01
Lobectomy	398 (23.2%)	1044 (28.0%)	
Total thyroidectomy	1303 (76.0%)	2666 (71.5%)	
Completion thyroidectomy	14 (0.8%)	21 (0.6%)	
Central neck dissection performed	550 (32.5%)	1289 (35.0%)	0.07
Lateral neck dissection performed	179 (10.6%)	344 (9.3%)	0.16
Recurrent laryngeal nerve transection	15 (0.9%)	35 (0.9%)	0.82
Operative time			< 0.01
<1 h	317 (18.5%)	223 (6.0%)	
1–2 h	885 (51.6%)	1975 (52.9%)	
2–3 h	288 (16.8%)	991 (26.6%)	
>3 h	225 (13.1%)	542 (14.5%)	
Length of stay			< 0.01
Same-day discharge	524 (30.6%)	1594 (42.7%)	
1 day	964 (56.2%)	1958 (52.5%)	
2 days	133 (7.8%)	109 (2.9%)	
>2 days	90 (5.2%)	63 (1.7%)	
Already inpatient	< 10 (0.2%)	< 10 (0.2%)	
Pathologic Characteristics			
Thyroid cancer histology			0.03
Papillary thyroid cancer	1482 (86.4%)	3124 (83.7%)	
Follicular thyroid cancer	137 (8.0%)	383 (10.3%)	
Oncocytic carcinoma	44 (2.6%)	92 (2.5%)	
Medullary thyroid cancer	34 (2.0%)	102 (2.7%)	
Poorly differentiated thyroid cancer/ anaplastic thyroid cancer	18 (1.0%)	30 (0.8%)	
Extent of cancer			< 0.01
Intrathyroidal	1609 (93.8%)	3544 (95.0%)	
Extrathyroidal	90 (5.2%)	123 (3.3%)	
Unknown	16 (0.9%)	64 (1.7%)	
N-stage			0.48
N0	638 (37.2%)	1366 (36.6%)	
N1	481 (28.0%)	1007 (27.0%)	
Nx	596 (34.8%)	1358 (36.4%)	
M-stage			< 0.01
M0	470 (27.4%)	822 (22.0%)	
M1	29 (1.7%)	37 (1.0%)	
Mx	1216 (70.9%)	2872 (77.0%)	

* Two-sided t-tests for continuous variables; chi-squared tests for categorical variables

Variables with < 10 patients are reported as < 10 to preserve anonymity

In univariate analysis, patients who underwent surgery by surgeons who routinely use IONM had preoperative compressive symptoms more often (19.9% vs. 15.2%, p < 0.01) and BMI > 40 (9.0% vs. 7.3%, p = 0.03) compared to those who underwent surgery by surgeons who did not use IONM routinely. The proportion of thyroid lobectomies was higher among patients undergoing surgery with routine use of IONM (28.0% vs. 23.2%, p < 0.01). Operative time was longer with routine use of IONM (p < 0.01) and the rate of same-day discharge was higher for patients operated on by surgeons who use IONM in ≥ 90% of cases (42.7% vs. 30.6%, p < 0.01). Compared to patients who underwent surgery without routine use of IONM, patients who underwent surgery with routine IONM use had lower rates of postoperative VC dysfunction in the short- (2.4% vs. 4.4%, p < 0.01) and long-term (2.2% vs. 3.7%, p < 0.01) (Table 2). Hypoparathyroidism events were less likely in the short- (6.5% vs. 10.7%, p < 0.01) and long-term (1.9% vs. 4.1%, p < 0.01) among patients who underwent surgery by surgeons who routinely use IONM compared to those who do not.

**Table 2:** Postoperative outcomes of patients who underwent thyroidectomy for thyroid cancer according to the surgeons’ IONM practice

	No Routine IONM use (n = 1,715)	Routine IONM use (n = 3,731)	*P* value*
Postoperative Outcomes			
Vocal Cord Dysfunction – short term (< 30 days)	75 (4.4%)	90 (2.4%)	< 0.01
Vocal Cord Dysfunction – long term (30–180 days)	64 (3.7%)	81 (2.2%)	< 0.01
Hypoparathyroidism – short term	183 (10.7%)	243 (6.5%)	< 0.01
Hypoparathyroidism – long term	71 (4.1%)	70 (1.9%)	< 0.01
ED visit postoperatively	65 (3.8%)	161 (4.3%)	0.37
30-day readmission	33 (1.9%)	62 (1.7%)	0.49
Hematoma requiring evacuation	11 (0.6%)	24 (0.6%)	0.99

* Two-sided t-tests for continuous variables; chi-squared tests for categorical variables

ED, emergency department

### Risk of vocal cord dysfunction

**Fig. 2:**
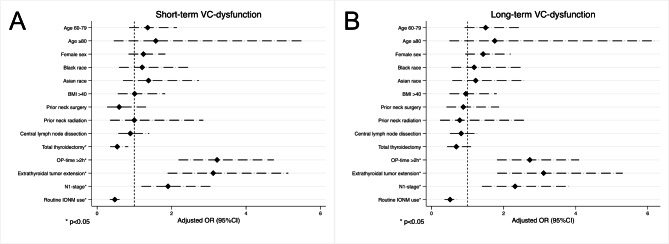
Forest plot of patient characteristics associated with postoperative short- (**A**) and long-term (**B**) vocal cord dysfunction among patients who underwent surgery for thyroid cancer Caption:*Multivariable logistic regression adjusted for: patient age, sex, race, BMI > 40, prior neck surgery prior neck radiation, extent of surgery, op-time, central and lateral LND, extrathyroidal extension, N-stage **Reference categories: Patient age < 40 years, male sex, white race, BMI ≤ 40, no prior neck surgery, no prior neck radiation, thyroid lobectomy, OP-time ≤ 2 h, thyroid surgery alone without central/lateral neck dissection, no extrathyroidal extension, N0-stage VC, vocal cord

After adjustment for demographic, clinical, and pathologic variables, routine use of IONM was associated with lower odds of postoperative VC dysfunction both in the short-term (aOR 0.48, 95%CI 0.34–0.66), p < 0.01) and long-term (aOR 0.52, 95%CI 0.36–0.74, p < 0.01) (Fig. 2). Extrathyroidal tumor extension and locoregional nodal metastases were independently associated with higher risk of postoperative VC dysfunction in the short-term (aOR 3.19, 95%CI 1.89–5.13, p < 0.01; aOR 1.91, 95%CI 1.19–3.05, p = 0.01, respectively) and long-term (aOR 3.11, 95%CI 1.82–5.31, p < 0.01; aOR 2.32, 95%CI 1.40–3.85, p < 0.01, respectively). A subgroup analysis in patients undergoing thyroidectomy with central neck dissection showed that routine use of IONM was associated with a lower likelihood of postoperative VC dysfunction, both in the short- (aOR 0.38, 95%CI 0.23–0.62, p < 0.01) and long-term (aOR 0.48, 95%CI 0.28–0.81, p < 0.01) (Table 3).

**Table 3:** Adjusted odds (aOR)* of outcomes in the subgroup of patients following surgery with central neck dissection for thyroid cancer with routine use of IONM (n = 1,839)

Variable	aOR (95% CI)	*P* value
Vocal cord dysfunction short-term	0.38 (0.23–0.62)	< 0.01
Vocal cord dysfunction long-term	0.48 (0.28–0.81)	< 0.01
Hypoparathyroidism concern – short term	0.68 (0.50–0.93)	0.02
Hypoparathyroidism concern – long term	0.45 (0.28–0.70)	< 0.01
Same-day discharge	1.25 (0.95–1.63)	0.11
ED visit postoperatively	1.40 (0.86–2.25)	0.17
30-day readmission	0.98 (0.50–1.92)	0.96
Hematoma	0.51 (0.19–1.39)	0.19

*Multivariable logistic regressions adjusted for: patient age, sex, race, BMI > 40, prior neck surgery, prior neck radiation, extent of surgery, op-time, extrathyroidal extension, N-stage

ED, emergency department

### Routine use of IONM

Routine use of IONM was associated with same-day discharge after surgery (aOR 2.03, 95%CI 1.76–2.34, p < 0.01) and a lower risk of postoperative hypoparathyroidism in the short- (aOR 0.67, 95%CI 0.53–0.83, p < 0.01) and long-term (aOR 0.54, 95%CI 0.37–0.78, p < 0.01). There were no significant differences in the likelihood of postoperative emergency department visits, 30-day readmission, or neck hematoma with or without routine IONM use (Table 4).

**Table 4:** Adjusted odds (aOR)* of outcomes following surgery for thyroid cancer with routine use of intraoperative nerve monitoring (IONM)

Variable	aOR (95% CI)	*P* value
Same-day discharge	2.03 (1.76–2.34)	< 0.01
Hypoparathyroidism concern – short term	0.67 (0.53–0.83)	< 0.01
Hypoparathyroidism concern – long term	0.54 (0.37–0.78)	< 0.01
ED visit postoperatively	1.20 (0.88–1.64)	0.24
30-day readmission	0.80 (0.51–1.25)	0.32
Hematoma	0.88 (0.39–1.72)	0.60

*Multivariable logistic regressions adjusted for: patient age, sex, race, BMI > 40, prior neck surgery, prior neck radiation, extent of surgery, op-time, central and lateral LND, extrathyroidal extension, N-stage

ED, emergency department

#### IONM use if surgeons did not use IONM routinely

**Fig. 3:**
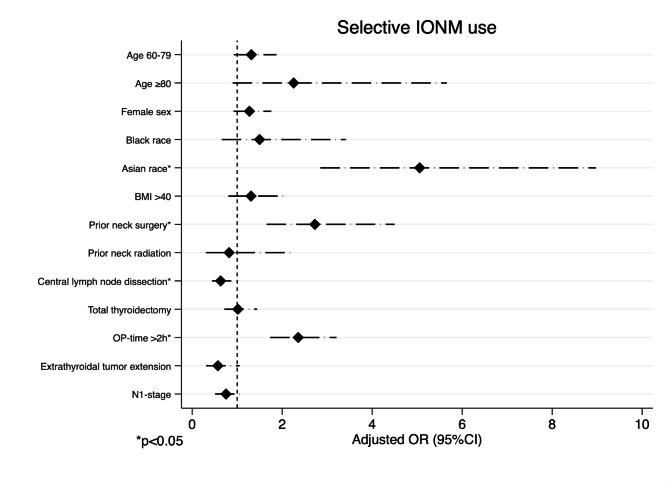
Forest plot of patient characteristics associated with selective IONM use if surgeons did not use IONM routinely Caption:*Multivariable logistic regression adjusted for: patient age, sex, race, BMI > 40, prior neck surgery prior neck radiation, extent of surgery, op-time, central and lateral LND, extrathyroidal extension, N-stage **Reference categories: Patient age < 40 years, male sex, white race, BMI ≤ 40, no prior neck surgery, no prior neck radiation, thyroid lobectomy, OP-time ≤ 2 h, thyroid surgery alone without central/lateral neck dissection, no extrathyroidal extension, N0-stage IONM, intraoperative nerve monitoring

In the subgroup of patients that had surgery by surgeons who did not use IONM routinely (N = 1,715), IONM was associated with unknown race, Asian race, and Hispanic ethnicity compared to non-hispanic white patients (aOR 8.62, 95%CI 6.09–12.22, p < 0.01; aOR 4.02, 95%CI 2.88–5.61, p < 0.01, aOR 5.06, 95%CI 2.85–8.97, p < 0.01, respectively), prior neck surgery (aOR 2.73, 95%CI 1.65–4.50, p < 0.01) and operative time > 2 h (aOR 2.63, 95%CI 1.73–3.21, p < 0.01) (Fig. 3). IONM was less likely to be used in patients undergoing central neck dissection (aOR 0.63, 95%CI 0.44–0.91, p = 0.02) and in patients with extrathyroidal tumor extension (aOR 0.53, 95%CI 0.39–0.74, p < 0.01). Patients that had surgery by surgeons who do not routinely use IONM were more likely to be discharged the day of surgery if IONM was used during thyroid surgery (aOR 1.85, 95%CI 1.29-0.67, p < 0.01) (Table 5). There were no significant differences in the likelihood of postoperative VC dysfunction, hypoparathyroidism events, emergency department visits, and 30-day readmissions with or without IONM in this subgroup of patients.

**Table 5:** Adjusted odds (aOR)* of outcomes with IONM use in the subgroup of patients who underwent thyroid surgery by surgeons who do not routinely use IONM (n = 1,715)

Variable	aOR (95% CI)	*P* value
Vocal cord dysfunction short-term	1.44 (0.81–2.58)	0.22
Vocal cord dysfunction long-term	0.91 (0.46–1.81)	0.79
Hypoparathyroidism concern – short term	0.96 (0.64–1.46)	0.86
Hypoparathyroidism concern – long term	1.01 (0.53–1.93)	0.98
Same-day discharge	1.85 (1.29-0.67)	< 0.01
ED visit postoperatively	1.05 (0.54–2.06)	0.89
30-day readmission	0.61 (0.21–1.77)	0.36
Hematoma	NA	NA

*Multivariable logistic regressions adjusted for: patient age, sex, race, BMI > 40, prior neck surgery, prior neck radiation, extent of surgery, op-time, central and lateral LND, extrathyroidal extension, N-stage

ED, emergency department

### Sensitivity analyses

Sensitivity analyses included uni- and multivariable adjusted analyses with cut-off values of 80% and 75% defining routine use of IONM. Results with these cut-off values were similar to those presented above. In addition, the distribution of IONM use among surgeons was binary, either high or low use. In the group of surgeons who had a high IONM use practice, the vast majority of surgeons use IONM in ≥ 90% of the cases. Therefore, this was the value used in defining surgeons who had routine IONM use.

## Discussion

In this contemporary cohort analysis of patients with thyroid cancer, the routine use of IONM by specialized thyroid surgeons was independently associated with a lower risk of both short- and long-term laryngoscopy-confirmed VC dysfunction. Routine IONM use was also associated with a higher likelihood of same-day discharge after surgery and a lower risk of hypoparathyroidism events. Among patients with thyroid cancer, extrathyroidal extension and locoregional nodal metastases were associated with a higher risk of VC dysfunction. IONM was not associated with a lower likelihood of VC dysfunction and hypoparathyroidism events if surgeons did not use IONM routinely.

Although IONM was developed to help reduce the risk of RLN injury, prior literature has failed to consistently demonstrate a lower risk of postoperative VC dysfunction with the use of IONM. Dralle et al. prospectively evaluated 16,448 consecutive thyroidectomies at 63 hospitals in Germany and found no statistically significant difference between the frequency of RLN injuries after visual RLN identification compared to the use of IONM [6]. In a systematic review and meta-analysis of studies comparing IONM to visual nerve identification alone, Pisanu et al. pooled effects from 23,512 patients across 20 studies and found no evidence of a difference in RLN palsy (overall, transient, or permanent) between groups [13]. Another meta-analysis of eight randomized controlled trials, with 4,977 nerves at risk, could not demonstrate a reduced risk of RLN injuries with the use of IONM compared to RLN visualization alone [14].

In contrast, other studies have suggested that IONM may be protective against RLN injury. The randomized clinical trial by Barczynski et al. demonstrated that RLN visualization with additional IONM was associated with a lower rate of VC dysfunction overall compared to nerve visualization alone (3.0% vs. 6.7%, p = 0.007), particularly in the group of patients undergoing thyroidectomy for cancer including central lymph node clearance, thyrotoxicosis, retrosternal or giant goiter, and thyroiditis [15]. In a 2016–2018 NSQIP analysis of 17,610 patients undergoing thyroidectomy for benign and malignant indications, Kim et al. found that IONM was associated with a lower risk of RLN injury [1]. Similarly, Leonard-Murali et al. performed an analysis of 9,527 patients who underwent thyroidectomy in the 2016–2017 NSQIP and found that IONM was associated with a lower risk of RLN injury (OR 0.83, 95%CI 0.69–0.98) [16]. This association was stronger among patients who underwent thyroidectomy specifically for thyroid cancer (OR 0.76, 95%CI 0.62–0.94). However, NSQIP defines RLN injury as symptomatic hoarseness and does not include data on whether the diagnosis was confirmed with laryngoscopy. The present study builds on existing work by demonstrating that routine use of IONM is associated with a lower risk of laryngoscopy-confirmed VC dysfunction in a cohort of patients with thyroid cancer, a group who, on average, are at increased risk of RLN injury during thyroidectomy [2, 6, 8, 16, 17]. The lower likelihood of postoperative VC dysfunction with routine use of IONM in the subgroup of patients who underwent central neck dissection suggests that routine IONM use may be particularly beneficial in patients with more advanced disease and/or more extensive surgery. Not surprisingly, tumors known to be more invasive, such as medullary, poorly differentiated, and anaplastic thyroid cancer, had a higher rate of postoperative VC dysfunction compared with differentiated thyroid cancer histologies. Since CESQIP contains data exclusively from specialized thyroid surgeons, it is possible that the benefit of routine IONM use is underestimated in CESQIP compared to NSQIP, which has a more heterogeneous patient and surgeon cohort.

The reasons for the low number of VC dysfunction in the short-term and/or the low recovery rate remain unclear. Unlike other prospective studies, e.g. the study by Schneider et al. where laryngoscopy was performed routinely on postoperative day 2, and in the follow-up 2, 4, and 6 months after surgery, laryngoscopy in this dataset was not performed on a specific day after surgery [18]. VC dysfunction in the short-term was confirmed with laryngoscopy within 30 days after surgery, as defined by CESQIP. It is possible that RLN palsies due to traction that resolve quickly after surgery may not have been detected if laryngoscopy was performed two to four weeks after surgery; this may explain the findings of similar low rate of VC dysfunction in the short- and long-term, and the low rate of detected recovery in this dataset. Additionally, follow-up may have been too short in some patients to diagnose recovery since it was not standardized when follow-up laryngoscopy should be performed; CESQIP only includes the information that laryngoscopy was performed between 31 and 180 days after surgery.

The missing association between IONM and postoperative VC dysfunction and other complications in the subgroup of patients operated on by surgeons who do not routinely use IONM suggests that there is limited benefit from this technique in the absence of standardization. Comparing two different time periods, before and after standardization of IONM, Chiang et al. demonstrated that standardizing IONM resulted in a decreasing rate of RLN palsy from 6.4 to 0.8% (p < 0.01) [11]. Another study comparing outcomes following thyroidectomies with and without standardized and routine use of IONM concluded that routine use of nerve monitoring allows less experienced surgeons to perform surgery safely with a similar RLN palsy rate as experienced surgeons [19]. Kuryga et al. examined 2351 nerves at risk and showed that surgeons trained in IONM had a significantly lower rate of RLN palsy compared to surgeons who were not trained and do not routinely use IONM (0.58% vs. 2.8%, p < 0.01) [20]. In our study, in the cohort of patients who underwent surgery by surgeons who did not routinely use IONM, nerve monitoring was associated with prior neck surgery, suggesting that IONM was then used selectively for anticipated difficult dissections. This may explain, in part, the missing association of IONM and VC dysfunction in this subgroup. However, because prior neck surgery was not associated with a higher likelihood of VC dysfunction in either this subgroup or the main cohort, we do not believe that this has a significant impact on the fact that IONM appears to be less beneficial when surgeons do not use it routinely.

The reason for the lower rate of hypoparathyroidism in patients undergoing thyroidectomy with routine IONM use is not entirely clear. One possible explanation could be that surgeons who use IONM routinely are more likely to also use other devices, such as vessel sealing devices or parathyroid visualization techniques, and to perform more careful dissections that result in less intraoperative bleeding or damage to surrounding tissue. Surgeon and hospital volume represent important contributors to postoperative complications. Adam et al. demonstrated that, on average, the threshold of > 25 total thyroidectomies per surgeon per year is independently associated with reduced complications [21]. The practice of IONM use may be one factor among many that could be separating two groups of surgeons. However, CESQIP is a dataset that is specifically designed to enroll high volume thyroid surgeons/institutions, so the differences among the members of this cohort may be more attenuated than for other, lower volume surgeons, and likely does not contribute meaningfully to the assessment of postoperative complications in this study. In addition, selective use of IONM in the group of surgeons who do not use IONM routinely was not associated with a significantly lower rate of postoperative VC dysfunction, therefore we believe the results of this study support the fact that IONM use is beneficial if surgeons are familiar with the technology.

In addition to reduced morbidity, routine use of IONM may be associated with population-level benefits, including decreased healthcare utilization and cost. A prior study using a Markov decision model concluded that IONM is cost-effective in the setting of thyroidectomy performed for benign disease if the rates of long-term VC dysfunction remain below 3.8% at one year [22]. In a similar study, Al-Quaryshi et al. concluded that IONM during planned total thyroidectomy for a 4.1 cm papillary thyroid cancer would be associated with an incremental cost-effectiveness ratio of US $46,427.97 per quality-adjusted life-year [23]. In their model, the assumption was that IONM would identify signal loss during planned total thyroidectomy, and the surgeon would be able to limit the operation to a thyroid lobectomy and avoid contralateral nerve injury. In another cost-effectiveness analysis, Rocke et al. concluded that although visual identification is the most cost-effective method, IONM can become cost-effective in selected cases if its use is associated with a reduction in risk of RLN injury rate of 50.4% [24]. Together, these studies suggest that IONM may be most cost-effective for high-risk cases, such as thyroidectomy for invasive thyroid cancers, however standardization and surgeon’s experience in IONM use is important.

Limitations of this study include its retrospective and observational nature. It is possible that there are coding errors in CESQIP. Some patients with both short- and long-term VC dysfunction may have been coded as long-term only. Not all patients underwent pre-operative laryngoscopy; it is possible that some patients may have had preoperative VC dysfunction that was only identified postoperatively. CESQIP does not provide details regarding the exact day after surgery when laryngoscopy was performed nor the reasons certain patients underwent laryngoscopy.

For cases where IONM was not used, it is not known whether the surgeon was able to visually identify the RLN. Therefore, the comparator group in this study may include patients both with and without visual nerve identification. Despite these limitations, CESQIP is uniquely suited to study IONM because it includes data from patients undergoing thyroidectomy by specialized thyroid surgeons and provides granular data on VC dysfunction confirmed via laryngoscopy and information on each surgeon’s IONM practice.

Given that routine use of IONM is associated with a lower risk of VC dysfunction, strategies are needed to understand barriers to the adoption of this technology. Although the majority of the surgeons in our cohort used IONM consistently, many did not use it or used it only selectively. Because our CESQIP cohort was comprised of specialized endocrine/thyroid surgeons, it is likely that use of IONM among non-specialized surgeons is lower. Previous NSQIP studies demonstrated IONM use rates of 62.7% and 63.9% overall, whereas 95% of recently fellowship-trained surgeons in the US used IONM in some or all of their cases [1, 4, 16]. For surgeons unfamiliar with this technology, streamlined training courses have been suggested by the International Neural Monitoring Study Group as a strategy to flatten the learning curve associated with IONM [25].

## Conclusion

In conclusion, the results of this CESQIP analysis demonstrate that the routine use of IONM by specialized thyroid surgeons during thyroidectomy for thyroid cancer is independently associated with a lower risk of RLN injury and hypoparathyroidism. These findings may be used to inform future guidelines’ recommendations on the appropriate use of IONM. Future studies are needed to identify barriers to broader IONM use.

## Data Availability

The datasets used and/or analyzed during the current study are available from the corresponding author on reasonable request.

## References

[1] Kim J, Graves CE, Jin C, Duh QY, Gosnell JE, Shen WT (2021). Intraoperative nerve monitoring is associated with a lower risk of recurrent laryngeal nerve injury: a national analysis of 17,610 patients. Am J Surg.

[2] Hayward NJ, Grodski S, Yeung M, Johnson WR, Serpell J (2013). Recurrent laryngeal nerve injury in thyroid surgery: a review. ANZ J Surg.

[3] SHEDD DP, BURGET GC (1966). Identification of the recurrent laryngeal nerve: electrical method for evaluation in the human. Arch Surg.

[4] Marti JL, Holm T, Randolph G (2016). Universal Use of intraoperative nerve monitoring by recently Fellowship-Trained thyroid surgeons is common, Associated with Higher Surgical volume, and impacts intraoperative decision-making. World J Surg.

[5] Abdelhamid A, Aspinall S (2021). Intraoperative nerve monitoring in thyroid surgery: analysis of United Kingdom registry of endocrine and thyroid surgery database. Br J Surg.

[6] Dralle H, Sekulla C, Haerting J, Timmermann W, Neumann HJ, Kruse E (2004). Risk factors of paralysis and functional outcome after recurrent laryngeal nerve monitoring in thyroid surgery. Surgery.

[7] Alesina PF, Rolfs T, Hommeltenberg S, Hinrichs J, Meier B, Mohmand W (2012). Intraoperative neuromonitoring does not reduce the incidence of recurrent laryngeal nerve palsy in thyroid reoperations: results of a retrospective comparative analysis. World J Surg.

[8] Chan WF, Lang BH, Lo CY (2006). The role of intraoperative neuromonitoring of recurrent laryngeal nerve during thyroidectomy: a comparative study on 1000 nerves at risk. Surgery.

[9] Haugen BR, Alexander EK, Bible KC, Doherty GM, Mandel SJ, Nikiforov YE (2016). 2015 american thyroid Association Management Guidelines for adult patients with thyroid nodules and differentiated thyroid Cancer: the american thyroid Association Guidelines Task Force on thyroid nodules and differentiated thyroid Cancer. Thyroid.

[10] NSQIP. PUF UserGuide 2020.

[11] Chiang FY, Lee KW, Chen HC, Chen HY, Lu IC, Kuo WR (2010). Standardization of intraoperative neuromonitoring of recurrent laryngeal nerve in thyroid operation. World J Surg.

[12] CESQIP. : CESQIP - database. https://cesqip.org/ Accessed.

[13] Pisanu A, Porceddu G, Podda M, Cois A, Uccheddu A (2014). Systematic review with meta-analysis of studies comparing intraoperative neuromonitoring of recurrent laryngeal nerves versus visualization alone during thyroidectomy. J Surg Res.

[14] Davey MG, Cleere EF, Lowery AJ, Kerin MJ (2022). Intraoperative recurrent laryngeal nerve monitoring versus visualisation alone - a systematic review and meta-analysis of randomized controlled trials. Am J Surg.

[15] Barczyński M, Konturek A, Cichoń S (2009). Randomized clinical trial of visualization versus neuromonitoring of recurrent laryngeal nerves during thyroidectomy. Br J Surg.

[16] Leonard-Murali S, Ivanics T, Nasser H, Tang A, Singer MC. Intraoperative nerve monitoring in Thyroidectomies for Malignancy: does it Matter? Am Surg. 2021;3134821991967. 10.1177/0003134821991967. 10.1177/0003134821991967 PMC8650166 33522279

[17] Lo CY, Kwok KF, Yuen PW (2000). A prospective evaluation of recurrent laryngeal nerve paralysis during thyroidectomy. Arch Surg.

[18] Schneider R, Randolph G, Dionigi G, Barczyński M, Chiang FY, Triponez F (2016). Prospective study of vocal fold function after loss of the neuromonitoring signal in thyroid surgery: the international neural monitoring Study Group’s POLT study. Laryngoscope.

[19] Alesina PF, Hinrichs J, Meier B, Cho EY, Bolli M, Walz MK (2014). Intraoperative neuromonitoring for surgical training in thyroid surgery: its routine use allows a safe operation instead of lack of experienced mentoring. World J Surg.

[20] Kuryga D, Wojskowicz P, Szymczuk J, Wojdyla A, Milewska AJ, Barczynski M (2021). Training in intraoperative neuromonitoring of recurrent laryngeal nerves reduces the risk of their injury during thyroid surgery. Arch Med Sci.

[21] Adam MA, Thomas S, Youngwirth L, Hyslop T, Reed SD, Scheri RP (2017). Is there a minimum number of Thyroidectomies a Surgeon should perform to optimize patient outcomes?. Ann Surg.

[22] Wang T, Kim HY, Wu CW, Rausei S, Sun H, Pergolizzi FP (2017). Analyzing cost-effectiveness of neural-monitoring in recurrent laryngeal nerve recovery course in thyroid surgery. Int J Surg.

[23] Al-Qurayshi Z, Kandil E, Randolph GW (2017). Cost-effectiveness of intraoperative nerve monitoring in avoidance of bilateral recurrent laryngeal nerve injury in patients undergoing total thyroidectomy. Br J Surg.

[24] Rocke DJ, Goldstein DP, de Almeida JR (2016). A cost-utility analysis of recurrent laryngeal nerve monitoring in the setting of total thyroidectomy. JAMA Otolaryngol Head Neck Surg.

[25] Wu CW, Randolph GW, Barczyński M, Schneider R, Chiang FY, Huang TY (2021). Training courses in laryngeal nerve monitoring in thyroid and parathyroid surgery- the INMSG Consensus Statement. Front Endocrinol (Lausanne).

